# WHO working group meeting to amend WHO Recommendations to assure the quality, safety and efficacy of live attenuated yellow fever vaccines

**DOI:** 10.1016/j.vaccine.2022.04.095

**Published:** 2022-06-09

**Authors:** Javier Martin, Alan David Thomas Barrett, Dianliang Lei, Philip Minor

**Affiliations:** a National Institute for Biological Standards and Control, London, United Kingdom; b University of Texas Medical Branch Sealy Institute for Vaccine Science, USA; c World Health Organization, Geneva, Switzerland; d St Alban, London, United Kingdom

**Keywords:** Yellow fever vaccines, Virus seed lot, Neurotropism testing, WHO Recommendations

## Abstract

The current WHO Recommendations to assure the quality, safety and efficacy of live attenuated yellow fever vaccines were adopted in 2010. This document recommends that vaccine virus master and working seed lots should be tested for viscerotropism, immunogenicity and neurovirulence in monkeys. A vaccine manufacturer has reported, recently, discrepancies on the clinical scoring of monkeys during assessment of working seed lots and suggested aligning neurotropism assessment of yellow fever vaccines virus seed lots with that of neurovirulence testing of polio vaccines virus seed lots. In this approach, clinical signs are recorded but do not form part of the assessment or pass/fail criteria. At its 71^st^ meeting in August 2020, the ECBS agreed to establish a drafting group and to consult with manufacturers and other stakeholders on the proposed amendment. Then a survey had been conducted to seek opinions of stakeholders on the neurotropism testing and revision of current WHO Recommendations for yellow fever vaccines. It was recognized from the answers of the survey that the test for neurovirulence in monkeys presents several technical challenges which could be addressed in the amended version of the Recommendations.

On 18–19 March 2021, a virtual WHO working group meeting was held to discuss a proposed draft of the amended text with participants of yellow fever vaccine manufacturers and relevant regulators. Overall, there was a consensus among manufacturers and regulators that clinical evaluation provides important information and should be retained as part of the neurotropism test. However, there was also agreement that the test is somewhat subjective, and that analysis can be difficult. It was recognized that there was potential for improvement in both test execution and analysis to increase harmonization between manufacturers. Alternative tests to the non-human primates neurovirulence test would be useful but it was agreed that none seem to be sufficiently developed at present. Based on these working group discussions, it was proposed that the appendix on neurotropism test to be further revised by the WHO drafting group and submitted to ECBS for review and adoption.

Issues other than neurotropism test were discussed in the meeting as well. There were a number of points identified during the meeting, such as new platform of production, animal models, deep sequencing, international standards, that are outside the current recommendations that are worthy of further discussion. Therefore, it is recommended that there would be a future meeting with various stakeholders to discuss the potential revision of the whole Recommendations for yellow fever vaccines in order to meet the current needs.

## 1 Introduction

The disease yellow fever is caused by yellow fever virus the prototype member of the genus Flavivirus. Yellow fever virus exists in a transmission cycle involving mosquitoes and primates, including humans. As with most infectious diseases, the majority of people infected with yellow fever virus will either be asymptomatic or have mild symptoms and completely recover. For people who develop symptoms, the time from infection until illness is typically 3 to 6 days. Clinical features include high temperature, severe headache, vomiting and muscle weakness. Up to 1 in 4 people will progress to a more severe form of the disease, the toxic phase, with serious symptoms such as yellow skin, bleeding, shock and organ failure. The case-fatality rate varies by outbreak and can be up to 50%. Yellow fever virus is currently endemic in 44 countries in Subsaharan Africa and Tropical South America and it is estimated that there are 109,000 cases of severe yellow fever and 51,000 deaths each year [1], [2].

Yellow fever virus was first isolated in 1927 by monkey to monkey passage of blood from Mr. Asibi, a Ghanaian man who had a mild case of yellow fever. Currently, the most effective control strategy for yellow fever is immunization with the live-attenuated 17D vaccine. The yellow fever virus 17D vaccine strain was derived from the wild-type Asibi strain by serial passages in chicken embryo tissue. Three substrains of the 17D vaccine virus are currently used for vaccine production in embryonated chicken eggs, namely 17D-204, 17DD and 17D-213. Sorbitol and gelatin are commonly used as stabilizers, and the vaccine preparation is lyophilized and kept under cold-chain conditions. The yellow fever vaccine is given as a single subcutaneous or intramuscular injection containing no less than 1000 international units (IUs) of antigen. Currently, there are five vaccine producers, globally, of which four have vaccines that are prequalified by the World Health Organization (WHO) (https://extranet.who.int/pqweb/vaccines/list-prequalified-vaccines). Some countries require an International Certificate of Vaccination against yellow fever for incoming visitors. Vaccination for this purpose is done in nationally authorized sites using WHO prequalified vaccines. Following WHO Strategic Advisory Group of Experts on immunizations recommendations, yellow fever vaccination is carried out to protect populations living or travelling in areas subject to endemic and epidemic disease, and to prevent international spread by minimizing the risk of importation of the virus by viraemic travellers [2].

As with all vaccines, there are detailed recommendations published by WHO to assure the quality, safety and efficacy of yellow fever 17D vaccine. These were first published in 1958 and the most recent update was published in WHO Technical Report Series in 2013 [3], and include testing for safety from viscerotropic and neurotropic disease in non-human primates (NHPs). Following a request from Sanofi Pasteur related to the yellow fever vaccine neurovirulence test in NHPs, a working group including experts from the National Regulatory Authorities (NRAs) of yellow fever vaccine producing countries, academia, other experienced NRAs, and representatives from industry was convened in a virtual meetings in March 2021.

## 2 Background to the meeting

Following consideration of the declaration of interests of participants according to WHO procedure and the announcement that its legal department had judged these not to be an impediment to all those present from participating in the meeting, Dr Dianliang Lei (WHO) proceeded to announce that the meeting would be chaired by Dr Philip Minor (WHO Consultant) and that Drs Alan Barrett (University of Texas Medical Branch) and Javier Martin (National Institute of Biological Standards and Control (NIBSC)) would act as rapporteurs.

The meeting remit was to understand the current regulatory practices for testing yellow fever vaccine seed lots, focusing on the neurovirulence test in NHPs, to seek opinions for the revision/amendment of the current WHO recommendations for yellow fever vaccines and to discuss any other issues related to the quality, safety and efficacy of yellow fever vaccines. All issues were discussed in the open session of the meeting with all participants present, although the final decision on the way forward towards developing the WHO Recommendations, its structure, scope and timetable were discussed in a closed session of the drafting group alone.

Drs Dianliang Lei (WHO) and Ivana Knezevic (WHO) gave a background to the current working group meeting. This included information on epidemiology and disease burden and details of available YF vaccines and WHO Technical Recommendations documents. Dr Knezevic gave an update on WHO Biological Standardization activities, which have recently required extraordinary efforts to support the COVID-19 response. She described the key role that WHO has played for 70 years in establishing both WHO written standards for the regulatory evaluation of vaccines in the form of WHO guidelines and recommendations to assure the quality, safety, and efficacy of biological products and measurement standards for vaccines and other biologicals such as International Standards and Biological Reference Materials necessary to standardize biological lot release assays. These norms and standards are based on scientific consensus achieved through international consultations and collaborative studies and designed to assist WHO Member States to ensure the quality and safety of biological products and related in vitro biological diagnostic tests worldwide. This work is accomplished through WHO’s biologicals programme coordinated by a Secretariat at WHO, Geneva, the WHO Expert Committee on Biological Standardization (ECBS) and WHO Collaborating Centres for Biological Standardization. Reports of the ECBS are published annually in the WHO Technical Report Series. Requirements for yellow fever vaccine were first formulated in 1958 and revised in 1975. Further revision of the Requirements was approved by ECBS in 1995. An Amendment to that annex was published in WHO TRS 964 (2012) and current Recommendations to assure the quality, safety and efficacy of live attenuated yellow fever vaccines were established in 2013 (TRS 978).

The current WHO Recommendations for yellow fever vaccines indicate that “Each virus master and working seed lot should be tested for viscerotropism, immunogenicity and neurotropism, in a group of 10 test monkeys” and sets up specifications for the neurotropism test using NHPs [3]. This test requires both clinical and histological evaluation producing scores that have to satisfy specific criteria in order for the virus seed lot to meet the requirement for neurotropism. However, following reported discrepancies in the clinical scoring of NHPs during assessment of working seed lots, Sanofi Pasteur had requested that the neurotropism assessment be aligned with that used during neurovirulence testing of oral live attenuated poliomyelitis vaccine (OPV) seed lots [4]. In this approach, clinical signs are recorded but do not form part of the assessment or pass/fail criteria. At its 71st meeting in August 2020, the ECBS agreed that amendment of the Recommendations for yellow fever vaccines should be considered [5]. Following a survey of yellow fever vaccine manufacturers and NRAs, a number of issues had emerged, including the use of different virus substrains for vaccine manufacture, technical challenges associated with neurovirulence testing in NHPs, the use of different reference viruses, and the prospect of yellow fever vaccine development based upon the use of new cell substrates. At present the Recommendations only encompass of live attenuated 17D produced and only produced in embryonated chicken eggs [3]. It was anticipated that work on the amended document would commence in early 2021 and following public consultation would be presented to the ECBS for its review in October 2021. Following ECBS recommendation, the main focus of this meeting was to review the current approach for testing master and working virus seed lots and discuss survey outcomes. The meeting also provided the opportunity for reviewing other issues that may require update.

Dr Minor gave an overview on yellow fever vaccines covering its history and highlighting the main issues associated with current vaccine testing. The main conclusion is that this is a very old and successful vaccine, often termed a “legacy vaccine”, so any change(s) is/are difficult. Methods were developed many years ago and some are not optimally standardised. Remaining issues include the fact that vaccines are manufactured using chicken eggs potentially susceptible to adventitious agents, which is a safety concern. For this reason, the use of Specific-Pathogen-Free (SPF) eggs is required. Safety is a matter of concern for yellow fever vaccines including vaccine associated neurotropic disease that has been of growing concern since 2000 and vaccine associated viscerotropic disease since 1975 or earlier; although evidence to date suggests the latter is related to the host genetics and not the virus. Current safety tests use NHPs, as this is the natural host of wild-type yellow fever virus, with criteria that are not well defined. Common methodology among producers is clearly optimum but it has never been subjected to a collaborative study in which different vaccine substrains are tested side by side. A potential WHO reference virus that was prepared in 1973 [6] behaves very differently to established vaccine seeds in the neurovirulence test in NHPs producing very low histological damage but higher viremia than the seeds, and so has limitations for its use. It was also pointed out that, expressing amounts of virus in international units (IUs) for all purposes, including measuring vaccine potency, seems to make more sense than using mouse LD_50_ values but changing the dose the vaccinee receives into IU was a little problematic. In this sense, it would be useful to conduct a dose ranging clinical trial of each producer’s vaccine to establish the required effective dose, which would also help with current efforts to lower vaccine doses and ensure increased vaccine availability. Participants agreed that these were limitations that should be ideally corrected and described some specific experiences in these assays. Recent studies have shown that all WHO prequalified vaccines can use dose sparing [7] although dosing levels might be different for different vaccines.

## 3 Neurovirulence test in non-human primates for yellow fever vaccines

Sanofi Pasteur described their concerns with the neurovirulence test in NHPs, particularly related to the clinical scoring component. As with all producers, vaccine seed lots are generated infrequently, and the company performs the test very rarely with several years between tests. Sanofi Pasteur proposed that further standardization of the histopathological analysis component of the test using existing and shareable materials is possible whereas standardization of the clinical scoring component is less feasible as it would require considerable animal studies. The company tested three yellow fever virus working seed lots alongside a new working seed lot using a vaccine lot as a reference. Discrepancies were observed on the clinical scoring results of two working seed lots, while the overall test package results were similar including phenotypic and genetic analyses. An investigation was conducted and found that the clinical evaluation was variable while viscerotropism and immunogenicity results gave expected results. Histological scoring met the acceptance criteria and appeared more reliable than clinical scoring. The difference of clinical scores for the reference preparation over two tests conducted in 2011 and 2018 provided evidence of the variability of the clinical scoring for a same lot. Sanofi Pasteur described two publications supporting these findings with variability been shown in clinical scores but consistent histological results closely reflecting neurotropism [8], [9]. Sanofi Pasteur highlighted the inherent difficulties to assign accurate scores in the NHP model, questioning the accuracy of clinical scoring, uneasiness to identify such clinical signs in NHPs and the potential bias involving tests with many animals. They pointed out that, by comparison, the monkey neurovirulence test for OPV only uses histological scoring for assessing neurovirulence and clinical signs have never been part of the assessment in the NHP test, while in the transgenic mouse test for OPV only clinical evaluation (proportion of paralysis) is performed. Unfortunately, experience and data have shown that clinical scoring is reliable only with a standardized animal model, which is not the case for NHPs, that displays individual genetic and phenotypic heterogeneity leading to high variability in expression of clinical signs. Sanofi Pasteur proposed considering alignment of the assessment of yellow fever vaccines using the NHP test to current WHO recommendations for the neurovirulence test for OPV, for which clinical signs are recorded but do not form part of the assessment or the pass/fail criteria.

Following Sanofi Pasteur concerns, a survey on neurovirulence testing for yellow fever virus seed lots was conducted among interested parties. The survey involved five manufacturers and their corresponding NRAs. Participants were asked to provide details of their test protocol, its history and plans for the next five years as well as national requirements for the test, which in all cases follow WHO recommendations. The survey asked the question whether or not they had found any discrepancy between the outcomes of clinical evidence of encephalitis and histological lesions, and, apart from Sanofi Pasteur most responders indicated that there was generally good correlation between clinical signs and the extent of histological lesions, although some discrepancies were recorded in some tests. In relation to Sanofi Pasteur’s proposal to modify the significance of clinical scores of the NHP test in WHO Recommendations, basing pass/fail decisions only on histological lesions, the overall opinion among the five 17D manufacturers was to retain the NHP neurovirulence test as it is currently written in the WHO recommendations. Three manufacturers wanted to retain the test as currently described while two manufacturers saw opportunities to improve the test mainly in the standardization of the clinical scoring process.

Following discussion of the survey results in the meeting, there was overall agreement among manufacturers and NRAs that clinical evaluation provides important information and should be kept as part of the test. However, there was also agreement that the test is somewhat subjective, and analysis can be difficult. All participants agreed that there is potential for improvement in both test execution and analysis to increase harmonization between organizations. Alternative tests to the NHP neurovirulence test would be useful but it was agreed that none seem to be sufficiently developed at present; Nevertheless, consistency of viral attributes can also be assessed throughout a comprehensive testing package that may consider genetic and phenotypic characterization; although it was mentioned that analysis of plaque morphology would not be suitable for all seeds, given the presence of mixed plaque sizes in some 17D seed lots; genome sequence analysis is potentially useful but would require further development and establishment of clear and reliable pass/fail criteria.

## 4 Other proposal for revision/amendment of recommendations for yellow fever vaccines

Participants had also been asked in the survey for suggestions on any other possible revisions of the current WHO Recommendations for yellow fever vaccines and some indicated the need for improvements in statistical analysis of the NHP neurovirulence test, the need to establish reference materials for potency assays, and possible changes in tests for genotype characterization and adventitious agents. Discussions on potential alternatives to the NHP neurovirulence test continued and the possible need to expand the WHO Recommendations to include production of cell-based live-attenuated yellow fever vaccines and vaccines using different platform technologies such as inactivation, RNA, DNA, viral vectors, VLPs, etc. was also discussed.

In order to help in the debate, Dr Alan Barrett (University of Texas Medical Branch) gave a comprehensive overview on current progress in research and development of yellow fever vaccines covering animal models, correlates/surrogates of protection and new cell substrates/platforms for vaccine production. Currently, only NHPs are approved as animal models for yellow fever vaccines. There is potential for mouse models to be applied to yellow fever vaccine quality control, but it is currently only a research tool. At present, 17D is the only yellow fever virus seed and embryonated chicken eggs is the only cell substrate approved for yellow fever vaccine production in WHO recommendations. WHO Recommendations would need to be changed if different virus seeds/platform technologies were to be used to produce vaccine. Possible improvements in the NHP neurovirulence test include the use of telemetry but this does not appear to have been applied to 17D vaccine studies and will require investigation as a tool for measuring clinical signs. Establishing international norms and standards would be required if dose sparing is to be implemented and we will need a better understanding of neutralizing antibody titers as a correlate/surrogate of protection for all 17D vaccines. The use of deep sequencing as an alternative to tests in animals shows a lot of potential for the quality control of yellow fever vaccines as there is high consistency between vaccine seeds and products but standardizing the procedure and establishing appropriate pass/fail criteria is challenging at the present time.

## 5 Conclusions, recommendations to WHO

In summary, regarding the neurotropism testing of the yellow fever vaccine virus seed lots, there was a consensus among manufacturers and NRAs that clinical evaluation provides important information and should be retained as part of the neurotropism test. However, there was also agreement that the test is somewhat subjective, and that analysis can be difficult. It was recognized that there was potential for improvement in both test execution and analysis of test outcomes to increase harmonization between organizations. Based on the above agreements, it was concluded that the drafting group would make suggestions and revision for future improvements to the NHP neurovirulence test in the appendix 2 of the Recommendations to assure the quality safety and efficacy of live attenuated yellow fever vaccines to reduce the potential of subjective impact on the results of neurovirulent test in NHP. The revised appendix will be circulated among the working group for comments with an aim to submit a final version to ECBS for adoption. There were a number of points identified, such as new platform of production, animal models, deep sequencing, international standards, during the meeting outside the current recommendations that are worthy of further discussion, so it is recommended there would be a future meeting with various stakeholders to discuss other potential vaccine candidates that do not fit the current recommendations and whether or not the recommendations as a whole need redrafting.

Disclaimer

The author is staff member of the World Health Organization. The authors alone are responsible for the views expressed in this article and they do not necessarily represent the decisions, policy or views of the World Health Organization.
